# The circadian clock in the piriform cortex intrinsically tunes daily changes of odor-evoked neural activity

**DOI:** 10.1038/s42003-023-04691-8

**Published:** 2023-03-27

**Authors:** Shunsuke Takeuchi, Kimiko Shimizu, Yoshitaka Fukada, Kazuo Emoto

**Affiliations:** 1 Department of Biological Sciences, Graduate School of Science, The University of Tokyo, 7-3-1 Hongo, Bunkyo-ku Tokyo, 113-0033 Japan; 2 Department of Pathological Cell Biology, Medical Research Institute, Tokyo Medical and Dental University, 1-5-45 Yushima, Bunkyo-ku Tokyo, 113-8510 Japan; 3 Laboratory of Animal Resources, Center for Disease Biology and Integrative Medicine, Graduate School of Medicine, The University of Tokyo, 7-3-1 Hongo, Bunkyo-ku Tokyo, 113-0033 Japan; 4 International Research Center for Neurointelligence (WPI-IRCN), The University of Tokyo, 7-3-1 Hongo, Bunkyo-ku Tokyo, 113-0033 Japan

**Keywords:** Neural circuits, Cellular neuroscience, Neuronal development

## Abstract

The daily activity in the brain is typically fine-tuned by the circadian clock in the local neurons as well as by the master circadian clock in the suprachiasmatic nucleus (SCN) of the hypothalamus. In the olfactory response, odor-evoked activity in the piriform cortex (PC) and olfactory behavior retain circadian rhythmicity in the absence of the SCN, yet how the circadian rhythm in the PC is achieved independently of the SCN remains elusive. Here, to define neurons regulating the circadian rhythm of the odor-evoked activity in the PC, we knocked out the clock gene *Bmal1* in a host of specific neurons along the olfactory circuit. We discovered that *Bmal1* knockout in the PC largely abolishes the circadian rhythm of the odor-evoked activity. We further showed that isolated PC exhibits sustained circadian rhythms of the clock gene *Per2* expression. Quantitative PCR analysis revealed that expression patterns of multiple genes involved in neural activity and synaptic transmission exhibit circadian rhythm in the PC in a BMAL1-dependent manner. Our findings indicate that BMAL1 acts intrinsically in the PC to control the circadian rhythm of the odor-evoked activity in the PC, possibly through regulating expression patterns of multiple genes involved in neural activity and transmission.

## Abstract (web summary)

Loss of the clock gene, *Bmal1*, in the piriform cortex largely abolishes the circadian rhythm of odor-evoked activity in mice, suggesting a broader regulatory role for Bmal1 in olfactory neural activity and transmission.

## Introduction

Many organs including the brain exhibit circadian rhythms in their biological processes. Recent physiological and behavioral studies showed that the circadian rhythmicity is observed in multiple neural functions including memory & learning, emotional response, and sensory activity such as audition and olfaction^1–4^. These circadian rhythms of neural activities are typically generated and maintained through a series of transcription factors called clock genes. The transcription/translation levels of these clock genes oscillate in a 24-h cycle, via the negative transcription-translation feedback loop regulation^5–7^. Indeed, knocking out the core clock genes such as *Bmal1* and *Period1/2* (*Per1/2*) largely abolishes the circadian rhythms of neural activities^8,9^, underscoring their crucial roles in the circadian rhythms of neural activities.

In mammals, the suprachiasmatic nucleus (SCN) of the hypothalamus is widely believed to be the master circadian oscillator orchestrating the clock of the entire body^10–12^. Consistently, the core clock genes are abundantly expressed in the SCN^13,14^. On the other hand, a growing body of evidence suggests that the core clock genes are widely expressed in virtually all cells of the entire body, and that certain neural activities show circadian rhythms independent of the SCN^15,16^. One such example is the olfactory circuit, in which odor information is initially detected by the olfactory sensory neurons in the olfactory epithelium, then relayed to the mitral/tufted cells in the olfactory bulb, and eventually processed in the pyramidal neurons of the piriform cortex (PC)^17,18^. Odor-evoked activity in the PC shows a circadian rhythm with the highest response at night^1^, which is paralleled by the olfactory discrimination accuracy^19,20^. This circadian rhythmicity of the odor-evoked activity in the PC is unaffected by surgical removal of the SCN, although the oscillation phase tends to be affected^1^. These data suggest that the circadian rhythmicity of the odor-evoked activity in the PC is generated by circadian oscillators outside of the SCN. Consistent with this notion, surgical removal of the olfactory bulb in mice partially disturbs the circadian rhythm of the odor-evoked activity in the PC^1^. It is, however, still unknown what neural populations in the olfactory circuit contributes to the circadian rhythm of the odor-evoked activity in the PC, partially due to less accessibility of the surgical approach.

In this study, by combining multiple Cre recombinase-expressing mice and adeno-associated virus (AAV) transfection, we knocked out the clock gene *Bmal1* in specific sets of neurons in the olfactory circuit. We show that BMAL1 in the PC is required for the circadian rhythm of the odor-evoked activity. Furthermore, the isolated PC ex vivo maintained the circadian oscillation of *Per2* expression, another core clock gene. Interestingly, we found that expression levels of multiple genes related to neural activity and synaptic transmission exhibit circadian rhythms in the PC in a BMAL1-dependent manner. These findings suggest that the intrinsic molecular clock in the PC is critical for the circadian rhythm of the odor-evoked neural activity, and hint at the possibility that the intrinsic clock might exert such function through transcriptional regulation of the molecules related to neural activity and synaptic transmission.

## Results

### Odor-evoked neural activity in the PC exhibits a circadian rhythm

**Fig. 1:**
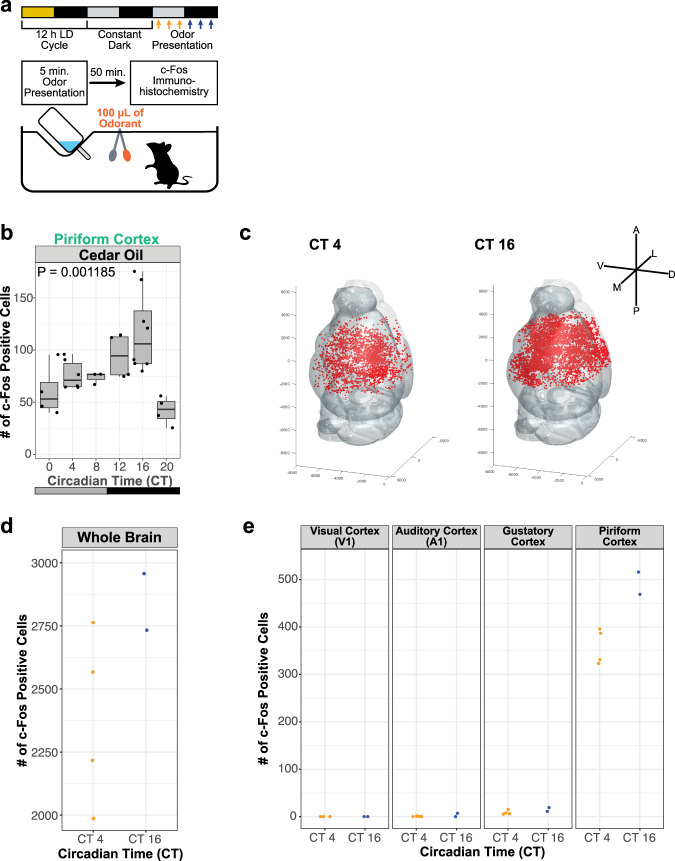
Circadian rhythms of neural activity are most evident in the PC. **a** Schematic illustration of the odor-presentation experiment for c-Fos quantification. 100 µL of cedar oil (1:1000 dilution in paraffin oil) was presented at six different timepoints for 5 min. **b** The number of c-Fos positive cells in the PC of mice exposed to the odor at six timepoints (CT0, 4, 8, 12, 16, 20). *N* = 4, 6, 3, 4, 8, 4 for CT0, CT4, CT8, CT12, CT16, CT20, respectively. *p* = 0.001185, one-way ANOVA. The line at the center of each boxplot depicts the median; the box depicts the third quartile and first quartile. **c** Representative whole-brain reconstruction of c-Fos positive cells in mice that were kept in home cages and then sacrificed at two timepoints (CT4, CT16). Red dots indicate individual c-Fos positive cells. **d** Quantification of the number of c-Fos positive cells in the whole brain. *N* = 4 and 2 for CT4 and CT16, respectively. **e** The number of c-Fos positive cells in five sensory cortical areas: visual cortex (V1), auditory cortex (A1), gustatory cortex, and PC in mice that were kept in home cages and then sacrificed at two timepoints (CT4, CT16). *N* = 4 and 2 for CT4 and CT16, respectively.

To investigate the circadian rhythm of the odor-evoked activity in the mouse brain, we presented cedar oil to the mouse as described previously^1^, and quantified the number of c-Fos positive cells^21^ in the PC for six timepoints throughout the day (Fig. 1a). Consistent with previous studies^1,22^, by comparing the number of c-Fos positive cells among six timepoints, we observed a peak at the subjective night CT16 (Fig. 1b, Supplementary Fig. 1a). Of note, the number of c-Fos positive cells showed no obvious correlation with other external conditions such as humidity and temperature (Supplementary Fig. 1b), suggesting that the circadian rhythm of odor-evoked PC activity is unrelated to external stimuli, but rather regulated through an internal mechanism. Additionally, mice exposed to another neutral odorant limonene(-) showed a significantly higher number of c-Fos positive cells in the PC at CT16 compared to CT4 (Supplementary Fig. 1c), comparable to the response to cedar oil. Thus, the circadian rhythm of the odor-evoked activity in the PC is not specific to cedar oil.

We next assessed whether odor-evoked neural activity might specifically fluctuate in the PC. To this end, we collected brain samples from subjective day CT4 and subjective night CT16 and quantified the number of c-Fos positive cells in serial sections spanning the whole brain. We found that the total number of c-Fos positive cells was higher at CT16 compared to CT4 (Fig. 1c, d). We further quantified the number of c-Fos positive cells in each sensory cortex by mapping the c-Fos positive cells onto a reference mouse brain of the Allen Brain institute three-dimensionally^23^. As a result, while the PC showed a higher number of c-Fos positive cells at CT16 (Fig. 1e), the number of c-Fos positive cells was scarce both at CT4 and CT16 in the primary visual cortex (V1), primary auditory cortex (A1) and gustatory cortex (Fig. 1e). The circadian fluctuation of neural activity is thus most obvious in the PC among sensory cortices. Interestingly, we found multiple brain areas in which c-Fos-positive cells were significantly higher at CT16 compared to CT4 including the somatomotor cortex, cortical subplate, basolateral amygdala, and hypothalamus (Supplementary Fig. 1d).

### Circadian rhythmicity of the odor-evoked activity in the PC are intrinsically maintained independently of the SCN

**Fig. 2:**
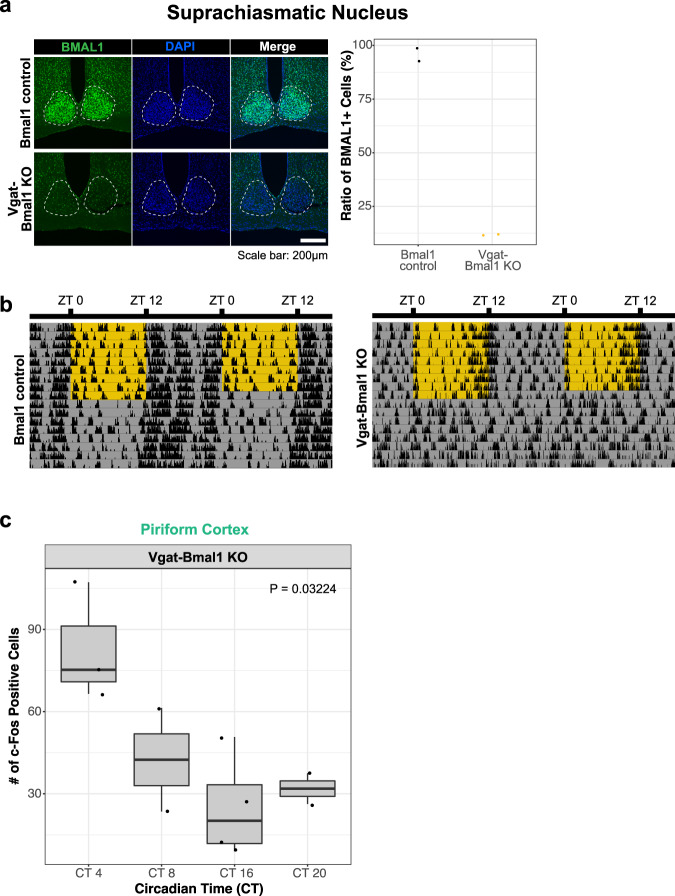
Bmal1 KO in the SCN fails to influence the circadian rhythmicity of the odor-evoked neural activity in the PC. **a** Representative images of the suprachiasmatic nucleus (SCN) of Bmal1-control mice and Vgat-Bmal1 KO mice, immunohistochemically stained with BMAL1 antibody. The ratios of BMAL1 + cells over DAPI + cells were quantified in the SCN. **b** Representative locomotor activity patterns of Bmal1-control mice and Vgat-Bmal1 KO mice. Yellow shades and gray shades indicate light ON and OFF respectively. **c** Quantification of c-Fos positive cells in the PC of Vgat-Bmal1 KO mice which underwent odorant exposure identical to Fig. 5A. *N* = 3, 2, 4, 2 for CT4, CT8, CT16, CT20, respectively. *p* = 0.03224, one-way ANOVA. The line at the center of each boxplot depicts the median; the box depicts the third quartile and first quartile.

Circadian rhythms of neural activities are widely thought to be generated by the SCN^10–12^ and the clock genes such as *Bmal1*^8,9^. While a previous report has suggested that the circadian rhythm of the odor-evoked activity in the PC operates independent of the SCN^1^, this report solely relied on surgical removal of the SCN, which could be confounded by inevitable damages to other brain structures. We thus sought to test whether BMAL1 expression in the SCN contributes to the circadian rhythm of the odor-evoked activity in the PC with less invasive methodologies. To this end, we took advantage of the evidence that the SCN is mostly comprised of GABAergic neurons, and that GABAergic neurons are required for the circadian rhythm of SCN activity^24^. Specifically, we conditionally knocked out the clock gene *Bmal1* in GABAergic neurons by crossing Vgat-Cre mice^25^ against Bmal1-floxed mice in which loxP sequences are inserted in the coding region of the *Bmal1* gene^26^. As expected, the number of BMAL1-positive cells in the SCN was significantly reduced in these conditional KO mice (“Vgat-Bmal1 KO mice”) compared to control mice (Fig. 2a). While control mice showed a clear circadian rhythm in locomotor activity both under the LD condition (light condition is indicated by yellow-shaded area) and the following DD condition (Fig. 2b, left), Vgat-Bmal1 KO mice failed to show a circadian rhythm under the DD condition (Fig. 2b, right), suggesting that the circadian rhythm in the SCN was effectively disrupted in Vgat-Bmal1 KO mice. By contrast, the number of c-Fos positive cells in the PC following cedar oil presentation retained circadian rhythmicity (Fig. 2c), suggesting that BMAL1 expression in GABAergic SCN neurons is dispensable for the circadian rhythmicity of the odor-evoked activity in the PC. We note that the number of c-Fos positive cells peaked at CT4 in Vgat-Bmal1 KO mice while this number peaked at CT16 in control mice (Fig. 1d), consistent with a previous study in which SCN was surgically removed^1^. These data suggests that BMAL1 in GABAergic SCN neurons is dispensable for rhythm generation but might contribute to phase control of the circadian rhythm of the odor-evoked activity in the PC.

### *Bmal1* KO in neurons containing GCs and PC disrupts the circadian rhythm of the odor-evoked activity in the PC

**Fig. 3:**
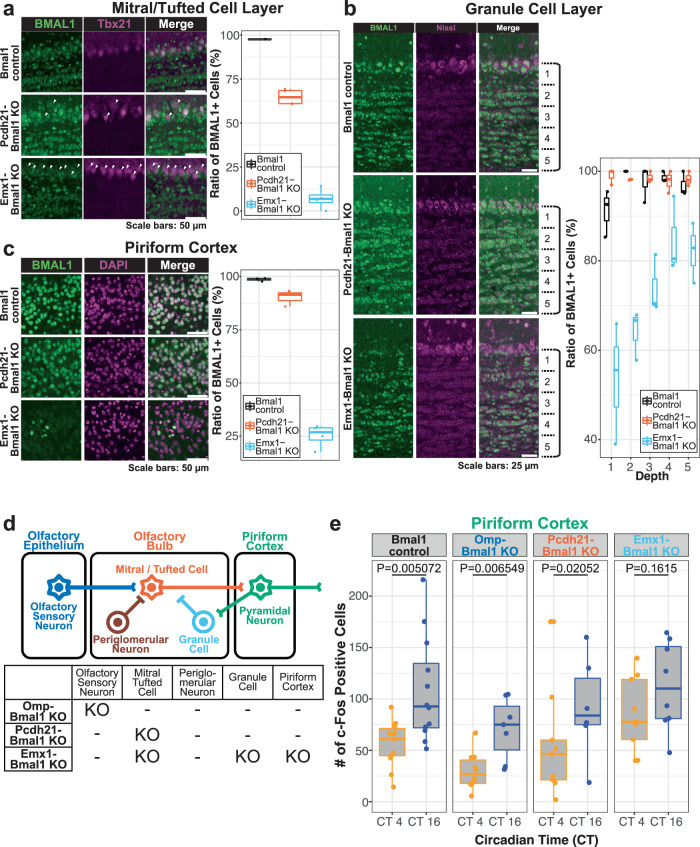
Bmal1 KO in GCs and PC partially disrupt the circadian rhythm of the odor-evoked activity in the PC. **a** Representative images of the mitral/tufted cell layer in the olfactory bulb of *Bmal1-floxed*^*fl/fl*^ mice (Bmal1-control), *Pcdh21-CreER*^*Tg/+*^*; Bmal1-floxed*^*fl/fl*^ mice (Pcdh21-Bmal1 KO) and *Emx1-Cre*^*Tg/+*^*; Bmal1-floxed*^*fl/fl*^ mice (Emx1-Bmal1 KO) immunohistochemically stained with BMAL1 antibody and mitral/tufted cell marker Tbx21 antibody. Arrowheads indicate BMAL1-; Tbx21+ cells. The ratios of BMAL1 + cells over Tbx21+ cells were 97.6%, 64.9 ± 2.29%, 7.1 ± 5.85% (mean ± standard deviation), in Bmal1-control mice, Pcdh21-Bmal1 KO mice, and Emx1-Bmal1 KO mice, respectively. The line at the center of each boxplot depicts the median; the box depicts the third quartile and first quartile. **b** Representative images of the granule cell layer in the olfactory bulb of Bmal1-control mice, Pcdh21-Bmal1 KO mice and Emx1-Bmal1 KO mice immunohistochemically stained with BMAL1 antibody and neuron marker Nissl. The ratio of BMAL1 + cells over Nissl+ cells were quantified in granule cell layers divided into five layers. *N* = 3 for each genotype, 91.1 ± 5.24, 99.0 ± 1.75, 53.5 ± 13.6%; 100.0 ± 0.00, 98.1 ± 0.09, 64.1 ± 5.53%; 97.7 ± 4.03, 98.6 ± 1.23, 73.9 ± 6.52%; 98.6 ± 1.18, 97.8 ± 2.68, 84.6 ±;8.55%; 96.9 ± 2.73, 98.3 ± 1.63, 82.1 ± 6.72% (mean ± standard deviation), in layers 1~5 of Bmal1-control mice, Pcdh21-Bmal1 KO mice, and Emx1-Bmal1 KO mice, respectively. The line at the center of each boxplot depicts the median; the box depicts the third quartile and first quartile. **c** Representative images of the PC in Bmal1-control mice, Pcdh21-Bmal1 KO mice, and Emx1-Bmal1 KO mice immunohistochemically stained with BMAL1 antibody and DAPI. The ratios of BMAL1 + cells over DAPI + cells were 98.6;± 0.69%, 90.2 ± 3.78%, 25.3 ± 5.43% (mean ± standard deviation), in Bmal1-control mice, Pcdh21-Bmal1 KO mice, and Emx1-Bmal1 KO mice, respectively. *N* = 4, 2, 4 for Bmal1-control mice, Pcdh21-Bmal1 KO mice, and Emx1-Bmal1 KO mice, respectively. The line at the center of each boxplot depicts the median; the box depicts the third quartile and first quartile. **d** Summary of the regions where *Bmal1* was knocked-out in each Cre-line. In Omp-Bmal1KO mice, *Bmal1* was knocked out in olfactory sensory neurons. In Pcdh21-Bmal1 KO mice, *Bmal1* was knocked out in mitral/tufted cells. In Emx1-Bmal1 KO mice, *Bmal1* was knocked out in mitral/tufted cells, granule cells, and PC neurons. **e** The number of c-Fos positive cells in the PC of Bmal1-control mice, Omp-Bmal1 KO mice, Pcdh21-Bmal1 KO mice, Emx1-Bmal1 KO mice exposed to an odorant. For Bmal1-control mice, *N* = 13 each for CT4 and CT16. For Omp-Bmal1 KO mice, *N* = 11 and 9 for CT4 and CT16, respectively. For Pcdh21-Bmal1 KO mice, *N* = 11 and 8 for CT4 and CT16, respectively. For Emx1-Bmal1 KO mice, *N* = 9 each for CT4 and CT16. *p* = 0.005072, 0.006549, 0.02052, 0.1615 for Bmal1-control mice, Omp-Bmal1 KO mice, Pcdh21-Bmal1 KO mice, Emx1-Bmal1 KO mice, respectively. Wilcoxon rank-sum test with Bonferroni correction. The line at the center of each boxplot depicts the median; the box depicts the third quartile and first quartile.

Since we failed to detect contribution of BMAL1 in the SCN to the circadian rhythmicity of the odor-evoked activity of the PC, we next asked whether BMAL1 functions inside the olfactory circuit. To this end, against *Bmal1*-floxed mice, we crossed multiple transgenic mice expressing Cre recombinase known to label specific neuronal populations along the olfactory circuit, including Omp-Cre mice^27^, Pcdh21-CreER mice^28^, and Emx1-Cre mice^29^. To check precisely where and how efficiently *Bmal1* was knocked out in these conditional KO mice (“Omp-Bmal1 KO mice”, “Pcdh21-Bmal1 KO mice”, “Emx1-Bmal1 KO mice”), we first conducted immunohistochemical analyses in the olfactory circuit including mitral/tufted cells, granule cells of the olfactory bulb, and the PC. In the mitral/tufted cell layer, we analyzed the ratio of BMAL1-positive cells against Tbx21-positive mitral/tufted cells and found that 97.6% of Tbx21-positive cells were BMAL1-positive in Bmal1-control mice while this ratio dropped to 64.9 ± 2.29% and 7.02 ± 5.85% in Pcdh21-Bmal1 KO mice and Emx1-Bmal1 KO mice, respectively (Fig. 3a). In the granule cell layer of the olfactory bulb, we analyzed the ratio of BMAL1-positive cells in five different depths since different granule cell types likely reside in the different depths^30,31^. As a result, while >90% of Nissl-positive neurons was BMAL1-positive in Bmal1-control mice and Pcdh21-Bmal1 KO mice, this ratio was reduced in all depths of the Emx1-Bmal1 KO mice (Fig. 3b). In the PC, while the ratio of BMAL1-positive cells was 98.6 ± 0.69% in Bmal1-control mice, this ratio was reduced to 90.2 ± 3.78% and 25.3 ± 5.40% in Pcdh21-Bmal1 KO mice and Emx1-Bmal1 KO mice, respectively (Fig. 3c). Thus, by utilizing the three Cre-lines, we successfully managed to knock out *Bmal1* in different neuronal subsets of the olfactory circuit (Fig. 3d).

We next presented cedar oil to these mice and quantified the number of c-Fos positive cells in the PC. First, in the control group where Cre recombinase is not expressed (Bmal1-control mice), the number of c-Fos positive cells in the PC was significantly higher at CT16 compared to CT4 (Fig. 3e). Similarly, the number of c-Fos positive cells in the PC was significantly higher at CT16 compared to CT4 both in Omp-Bmal1 KO mice and in Pcdh21-Bmal1 KO mice (Fig. 3e). By contrast, we observed no significant difference in the number of c-Fos positive cells in the PC between CT4 and CT16 in Emx1-Bmal1 KO mice (Fig. 3e). Further, Pcdh21-Bmal1 KO mice and Emx1-Bmal1 KO mice both showed rhythmic circadian locomotor activity in a constant dark condition (Supplementary Fig. 2), suggesting that the SCN-dependent circadian regulation was maintained in both KO mice. Given that *Bmal1* is knocked out in the mitral/tufted cells, granule cells, and the PC in Emx1-Bmal KO mice and that Pcdh21-Bmal1 KO mice in which *Bmal1* is knocked out in the mitral/tufted cells retained circadian rhythm of the odor-evoked activity in the PC, our data suggest that BMAL1 in the granule cells and/or the PC is required for the circadian rhythm of the odor-evoked activity in the PC.

### AAV-mediated PC-specific *Bmal1* KO abolishes the circadian rhythm of the odor-evoked activity in the PC

**Fig. 4:**
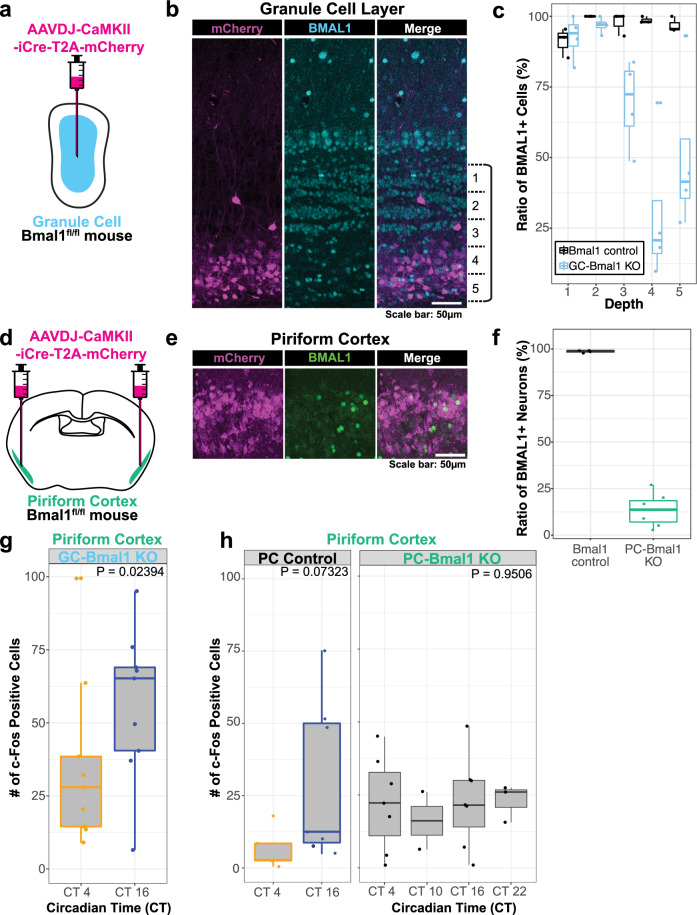
PC-specific Bmal1 KO abolishes the circadian rhythm of the odor-evoked activity. **a** AAVDJ-CaMKII-iCre-T2A-mCherry injection to the olfactory bulb (GC-Bmal1 KO). **b** Representative images of the granule cell layer in the olfactory bulb of GC-Bmal1 KO mice, immunohistochemically stained with BMAL1 antibody. **c** The ratio of BMAL1 + cells over Nissl+ cells were quantified in granule cell layers divided into five layers. For Bmal1-control mice: 91.1 ± 5.24%, 100.0 ± 0.00%, 97.7 ± 4.03%, 98.6 ± 1.18%, 96.9 ± 2.73%, (mean ± standard deviation), in layers 1~5, respectively. For GC-Bmal1 KO mice: 92.5 ± 7.81%, 96.8 ± 2.80%, 69.3 ± 15.8%, 17.1 ± 6.82%, 36.6 ± 8.85%, (mean ± standard deviation), in layers 1~5, respectively. *N* = 3 and 4 for Bmal1-control mice and GC-Bmal1 KO mice, respectively. The line at the center of each boxplot depicts the median; the box depicts the third quartile and first quartile. **d** AAVDJ-CaMKII-iCre-T2A-mCherry injection to the PC (PC-Bmal1 KO). **e** Representative images of the PC of PC-Bmal1 KO mice, immunohistochemically stained with BMAL1 antibody. **f** The ratios of BMAL1 + cells over Nissl+ cells were quantified in the PC. 98.6 ± 0.69%, 13.5 ± 8.59% (mean ± standard deviation) for Bmal1-control mice and PC-Bmal1 KO mice, respectively. *N* = 4 and 7 for Bmal-control mice and PC-Bmal1 KO mice, respectively. The line at the center of each boxplot depicts the median; the box depicts the third quartile and first quartile. **g** The number of c-Fos positive cells in the PC of olfactory bulb-injected granule cell conditional KO mice exposed to an odorant. *N* = 9 each for CT4 and CT16. *p* = 0.02394, Wilcoxon rank-sum test. The line at the center of each boxplot depicts the median; the box depicts the third quartile and first quartile. **h** The number of c-Fos positive cells in the PC of PC-injected with AAVDJ-CaMKII-mCherry (PC Control) and PC conditional KO mice (PC-Bmal1 KO) exposed to an odorant. For PC Control, *N* = 4 and 6 for CT4 and CT16, respectively. *p* = 0.07323, one-way ANOVA. For PC-Bmal1 KO, *N* = 7, 2, 7, 3 for CT4, CT10, CT16, CT22, respectively. *p* = 0.9506, one-way ANOVA. The line at the center of each boxplot depicts the median; the box depicts the third quartile and first quartile.

To further define the neural population required for the circadian rhythm of the odor-evoked activity in the PC, we limited *Bmal1* KO into a specific population of the olfactory circuit by expressing Cre recombinase via adeno-associated virus (AAV) injection. Specifically, we generated AAV where Cre recombinase and red fluorescent protein mCherry are simultaneously expressed downstream of the CaMKIIα promoter. CaMKIIα is known to be expressed both in the granule cells of the olfactory bulb and in the pyramidal neurons of the PC^32–34^. When we injected this AAV to the granule cell layers of the olfactory bulb (Fig. 4a), mCherry expression was largely observed in the deeper layers of the granule cell, and correspondingly BMAL1 expression was especially weak in the deeper layers (Fig. 4b, c, Supplementary Fig. 3a, b). We validated that mCherry is not expressed in cells outside of the granule cell layers, i.e. mitral/tufted cells (Supplementary Fig. 3a). When we injected the AAV to the PC (Fig. 4d), 55.5 ± 6.14% of Nissl-positive cells expressed mCherry (Supplementary Fig. 3c, d), and only 13.5 ± 8.59% of Nissl-positive cells expressed BMAL1 (Fig. 4e, f).

We next presented cedar oil to these mice and quantified the number of c-Fos positive cells in the PC. In mice where *Bmal1* was conditionally knocked out in granule cells (“GC-Bmal1 KO mice”), the number of c-Fos positive cells was significantly higher at CT16 compared to CT4 (Fig. 4g). On the other hand, in mice where *Bmal1* was knocked out in the PC (“PC-Bmal1 KO mice”), there was no significant difference in the number of c-Fos positive cells in the PC across four timepoints (Fig. 4h). Since the number of c-Fos positive cells was significantly higher at CT16 compared to CT4 in control group in which AAV-CaMKII-mCherry was injected to the PC (Fig. 4h), AAV injection alone has no significant effect on odor-evoked activity in the PC. These data suggest that the circadian rhythm of the odor-evoked activity in the PC was abolished in these mice. PC-Bmal1 KO mice showed rhythmic locomotor activity in a constant dark condition (Supplementary Fig. 3e), suggesting that the molecular clock in the SCN is functionally intact in PC-Bmal1 KO mice. These results indicate that BMAL1 expression in the CaMKIIα-positive pyramidal neurons of the PC is required for the circadian rhythm of the odor-evoked activity in the PC.

### PC can maintain circadian rhythm of clock gene expression in an autonomous manner

**Fig. 5:**
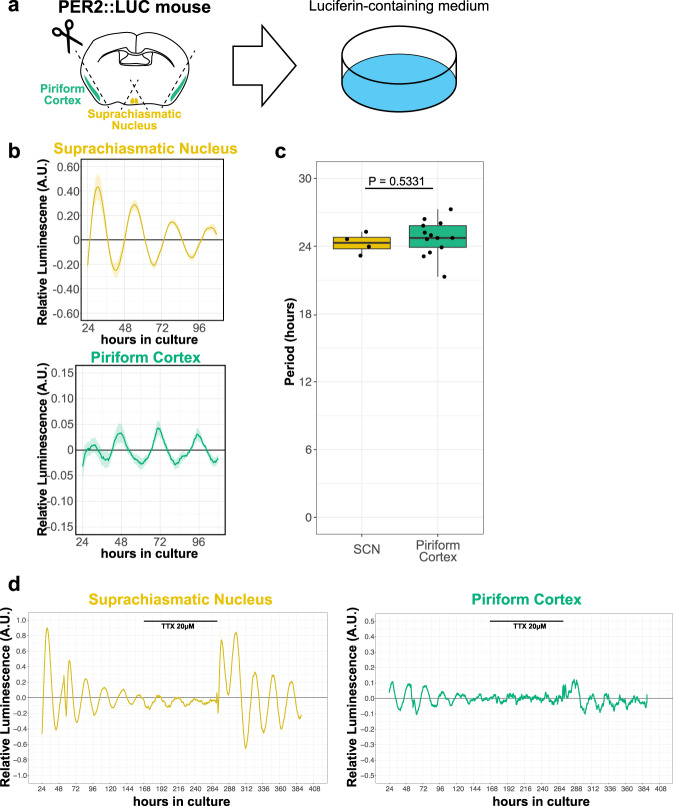
Clock gene expression in the PC retains circadian rhythmicity in the absence of SCN. **a** Schematic illustration of luciferase assay. The PC and suprachiasmatic nucleus were cut out from PER2::LUC mice and incubated in a luciferin-containing medium for up to 1 week. **b** Luminescence levels of the suprachiasmatic nucleus and the PC. Lines indicate the mean luminescence levels, and the shade indicates the mean ± standard error. **c** Mean period of the suprachiasmatic nucleus and PC. Periods were calculated by the Lomb-Scargle periodogram analysis. *N* = 4, 24.4 ± 1.10 h; *N* = 13, 24.73 ± 0.43 h, for the SCN and PC, respectively. *p* = 0.5331, Wilcoxon rank-sum test. The line at the center of each boxplot depicts the median; the box depicts the third quartile and first quartile. **d** Representative graphs of the luminescence levels of the SCN and the PC with TTX application.

Given that BMAL1 expression in the CaMKIIα-positive pyramidal neurons of the PC is responsible for the circadian rhythm of the odor-evoked activity in the PC, we reasoned that PC may be capable of self-sustaining the circadian rhythm of its clock gene expression. To test this possibility, we next conducted an ex vivo measurement of the circadian rhythm of the intrinsic clock using isolated tissues from PER2::LUC knock-in mice, in which the firefly *luciferase* cDNA is inserted immediately downstream of the *Per2* gene^35^. In brief, we generated acute slice sections of the PC and measured the luminescence level for up to 1 week in a luciferin-containing medium (Fig. 5a). As a positive control, we first confirmed that the SCN showed a sustained circadian rhythm of luminescence with a period of 24.4 ± 1.10 h (Fig. 5b) as reported previously^35^. Importantly, we observed sustained circadian rhythm in the PC with a period of 24.73 ± 0.43 h (Fig. 5b). There was no significant difference in the observed period between the SCN and the PC (Fig. 5c). To investigate whether action potential-based neural activity is necessary for the oscillation of clock gene expression in the PC, we applied tetrodotoxin (TTX) to the culture medium and found that the circadian rhythm damped out in the PC as well as the SCN and that the rhythm was re-activated by media change safter TTX treatment. These data suggest that neural activity is necessary for circadian oscillation of clock gene expression in the PC (Fig. 5d). Our findings thus indicate that the PC can intrinsically maintain the circadian rhythm of the clock gene expression in the absence of other brain regions.

### Genes related to neural activity show circadian rhythms in the PC in a BMAL1-dependent manner

**Fig. 6:**
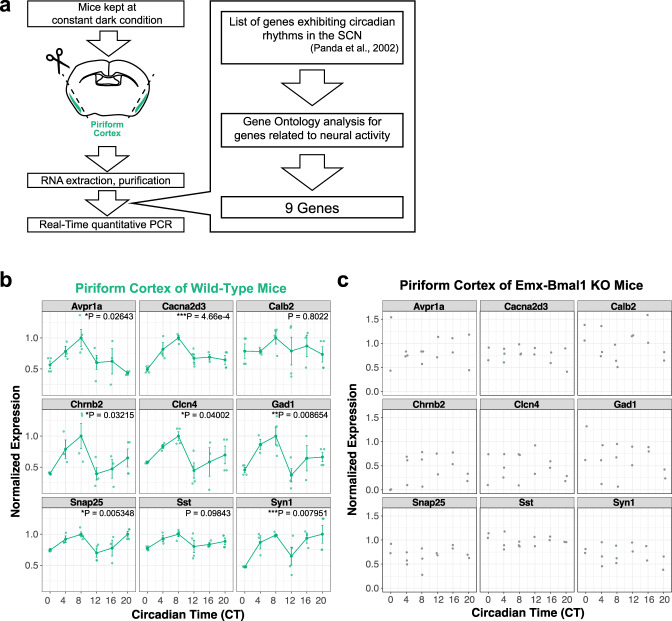
Genes related to neural activity show circadian rhythmicity in the PC. **a** Schematic illustration of real-time quantitative PCR. Mice were kept in 12-h Light/Dark condition for at least 3 weeks. Prior to sacrifice, mice underwent a constant dark condition, and PC and SCN were dissected for and RNA extraction, purification for real-time quantitative PCR. **b** Relative mRNA expression of *Avpr1a, Cacna2d3, Calb2, Chrnb2, Clcn4, Gad1, Snap25, Sst, Syn1* mRNA levels in the PC of wild-type mice. *N* = 4, 3, 4, 4, 3, 4 for CT0, CT4, CT8, CT12, CT16, CT20, respectively. One-way ANOVA with Bonferroni correction. Error bars indicate standard error. **c** Relative mRNA expression of *Avpr1a, Cacna2d3, Calb2, Chrnb2, Clcn4, Gad1, Snap25, Sst, Syn1* mRNA levels in the PC of Emx1-Bmal1 KO mice. *N* = 2, 3, 3, 2, 2, 2 for CT0, CT4, CT8, CT12, CT16, CT20, respectively.

Given that clock genes including *Bmal1* and *Per2* encode transcriptional regulators, the molecular clock in the PC is likely to regulate neural activity in a circadian manner through downstream gene expression. A previous study^36^ identified a set of genes acting downstream of the clock genes in the SCN, and we reasoned that at least some of these genes are likely to operate similarly in the PC. We thus extracted nine candidates from this set of genes deemed related to neural function through our gene ontology analyses and measured the mRNA levels of these genes in the PC at six timepoints throughout the day (Fig. 6a, Supplementary Table 1). We found that seven out of nine genes—*Avpr1a, Cacna2d3, Chrnb2, Clcn4, Gad1, Snap25, and SynI*—showed circadian rhythms of expression levels, and all seven peaked at CT8 (Fig. 6b). Next, we investigated whether the mRNA levels of these seven genes are regulated through BMAL1 transcription regulation. To this end, we collected the PC from Emx1-Bmal1 KO mice and quantified the mRNA levels of these genes. First, we confirmed that the mRNA levels of *Bmal1* were significantly suppressed compared to wild-type mice (Supplementary Fig. 4). Importantly, we failed to detect circadian rhythms in the seven genes that showed circadian rhythm in the wild-type mice (Fig. 6c), suggesting that the expression of these genes is transcriptionally regulated through BMAL1 in the PC. Taken together, our data suggest that BMAL1 in the PC regulates the expression patterns of multiple target genes related to neural function in a circadian manner, which could contribute to intrinsic control of the odor-evoked neural activity of the PC.

## Discussion

In this study, we focused on the mouse olfactory system and showed that BMAL1 in the pyramidal neurons of the PC intrinsically regulates the circadian rhythm of the odor-evoked neural activity. This notion is supported by the following lines of evidence. Firstly, the circadian rhythm of the odor-evoked neural activity was maintained in Vgat-Bmal1 KO mice in which BMAL1 expression was largely diminished in GABAergic neurons including over 90% of SCN neurons (Fig. 2), suggesting that the clock genes in GABAergic neurons in the SCN are dispensable for the odor-evoked neural activity in the PC. Secondly, *Bmal1* KO exclusively in the CaMKIIα-positive pyramidal neurons in the PC largely abolished the circadian rhythm of the odor-evoked neural activity in the PC (Figs. 3, 4). Lastly, isolated brain slices of the PC maintained the circadian rhythm of *Per2* expression at least for 3 days and this rhythm damped out upon TTX application (Fig. 5), suggesting that action potential-based neural activity likely contributes to intrinsic control of the clock gene expressions in the PC.

The present study showed an indispensable role of BMAL1 expression in the PC for the circadian rhythm of the odor-evoked activity in the PC. However, we could not clearly test the role of BMAL1 expression in the olfactory bulb because Pcdh21-Bmal1 KO removed BMAL1 from only ~30% of mitral/tufted cells (Fig. 3). Notably, a previous report suggested that the olfactory bulb functions as an independent circadian system regulating circadian rhythms of the odor-evoked neural activity, as surgical removal of the olfactory bulb abolished the circadian rhythms of the odor-evoked response in the PC^1^. Since the PC receives the vast majority of afferent fibers from the olfactory bulb^37,38^, it would be important in the future to understand how the olfactory bulb and the PC interact with each other to shape the circadian rhythms of the odor-evoked activity in the PC. Given that the mitral/tufted cells in the olfactory bulb project to the PC^37,38^, one potential scenario is that neural activity might contribute to synchronization between the olfactory bulb project and the PC.

The present study identified seven genes whose mRNA expressions showed circadian rhythms under the control of BMAL1 (Fig. 6). Of the seven genes identified, *Avpr1a* encodes a G-protein coupled arginine vasopressin receptor, and it increases intracellular Ca^2+^ levels to activate ERK/CREB^39^. *Chrnb2* encodes a subunit of a nicotinic acetylcholine receptor that promotes cation influx to depolarize the membrane potential^40^. *Clcn4* encodes a voltage-gated chloride channel. Hence the circadian rhythms of expressions of these genes may directly shape the circadian rhythm of the odor-evoked activity in the PC. Unexpectedly, other genes are associated with presynaptic, rather than postsynaptic, functions. *Cacna2d3* encodes a subunit of the voltage-gated calcium channel Ca_v_2.2^41^ which functions at presynaptic terminals^42–44^. Ca_v_2.2 channels are known to interact with the active zone proteins including SNAP25^45–48^. *SynI* encodes Synapsin I which contributes to the size control of the synaptic vesicle pool within the presynapses^49^. One possible scenario, therefore, is that BMAL1 in the PC regulates the expression of Ca_v_2.2, SNAP25, and Synapsin I to regulate the circadian rhythm of the odor-evoked output from the PC. Consistently, we found that action potential-based neural activity is required for in intrinsic circadian oscillation of clock gene *Per2* in the isolated SCN as well as the PC (Fig. 5d). However, we have to take into account that our experiments were limited to c-Fos induction, which does not directly measure neural activity. We would need to conduct other experiments such as calcium imaging and electrophysiological analyses to measure neural activity in a less indirect approach. For example, further investigations using electrophysiology and calcium imaging will be needed to understand how the local circadian clock indeed impacts on neural activity and synapse transmission in the PC.

Overall, we show in our current analyses that the molecular clockwork in the pyramidal neurons of the PC intrinsically regulates the circadian rhythm of the odor-evoked neural activity. We further show candidate genes that might contribute to the circadian rhythm of the odor-evoked neural activity under the control of the molecular clock. Since the molecular clock appears to target distinct genes in different tissues^50,51^, it is highly possible that additional genes might be transcriptionally regulated by BMAL1 and contribute to the circadian rhythms of the olfactory response in the PC. It will be thus important to understand the comprehensive sets of BMAL1-targeting genes in the PC and how the gene products could contribute to the intrinsic control of the circadian rhythm of neural activity.

## Methods

### Animals

All animal experiments had received approval from the Animal Care Committee at the University of Tokyo. C57BL/6 J wild-type mice were purchased from Japan SLC, Inc. Bmal1-floxed mice, Emx1-Cre mice, Vgat-Cre mice, and PER2::LUC mice were provided as a kind gift from Dr. Fukada, Omp-Cre mice, Pcdh21-CreER mice, and tdTomato-flox(Ai9) mice were purchased from The Jackson Laboratory. Animals were group-housed, up to four animals per cage, in a temperature (23 ± 1˚C) and humidity (50 ± 20%) controlled environment, in a 12 h light, 12 h dark cycle with *ad libitum* access to food and water, light on at 7:00 AM. Male mice were used in all experiments.

### Odor presentation paradigm

Adult male mice (2–3 months of age) were isolated to a new cage (Japan CLEA, CL-0113-1) 1 week prior to odor presentation with *ad libitum* access to food and water. Two days prior to odor presentation, mice were transferred to a new cage and underwent a constant dark condition for >24 . On the day of the experiment, 100 µL of odorant (cedar oil, limonene(-)) was applied to a cotton swab and placed on the cage lid for 5 min. 50 min after odor presentation, mice were perfused for further histological experiments.

### Immunohistochemistry

Mice were anesthetized with Isoflurane (Pfizer), then transcardially perfused with ice-cold 10 mL 0.1 M phosphate buffer (pH 7.4), followed by ice-cold 25 mL fixative (4% paraformaldehyde in 0.1 M phosphate buffer). Whole brains were dissected, immersed in ice-cold fixative for 6 h. Next, brains were immersed in 0.1 M PBS (pH 7.4) containing 20% sucrose for 6 h, then transferred to 0.1 M PBS (pH 7.4) containing 30% sucrose for 6 h. Forty µm-thick coronal sections were cut with a sliding microtome (Yamato Kohki). Cut sections were stored in an Anti-freezing buffer (25% glycerol (Nakalai Tesque), 30% ethylene glycol (Nakalai Tesque), 0.1 M PBS) at −30 °C.

Brain sections were incubated in blocking buffer (3% Normal Donkey Serum (Jackson Immuno Research Laboratories), 0.05% TritonX-100, PBS) for 2 h at room temperature. Next, sections were incubated in primary antibody (Anti-c-Fos Rabbit pAb, Calbiochem, 1:20,000 dilution in blocking buffer; Anti-BMAL1 Rabbit Polyclonal, Novus Biologicals, 1:1000 dilution in blocking buffer; Anti-Tbr1 Rabbit Polyclonal, Abcam, 1:1000 dilution in blocking buffer; anti-Tbx21 guinea pig, a gift from Y Yoshihara, 1:10,000 dilution in blocking buffer) for 3 days at 4 °C. After primary antibody incubation, sections were washed with PBST for 10 min, five times at room temperature. Next, sections were incubated with a second antibody (Alexa 488-conjugated donkey anti-rabbit, Invitrogen, 1:500 in blocking buffer). Afterward, sections were washed with PBST for 10 min, five times at room temperature. Finally, for nuclear staining, sections were incubated with Nissl (Invitrogen, 1:200 in PBS) or DAPI (Sigma-Aldrich, 1:2000 in PBS) for 1 h at room temperature. Sections were mounted with Prolong Diamond (Thermo Fisher Scientific) antifade.

### Microscopy and cell counting

For whole-brain 3D Mapping, brain sections were imaged by KEYENCE.

For other imaging, brain slices were imaged by confocal microscopy (Leica, SP8).

Brain sections immunostained with c-Fos antibody were quantified with the following procedures using ImageJ. First, the background signal intensity was calculated by averaging ten areas of the brain section without any cells. The background intensity was subtracted and despeckled. The signal threshold was set to 40, and the signals >30 µm were counted as c-Fos positive cells. For other imaging, brain slices were imaged by confocal microscopy (Leica, SP8).

### Locomotor activity analysis

Mice were housed individually, and their spontaneous locomotor activities were recorded using an area sensor (Elekit) with an infrared detector. Locomotor activity was collected every minute and analyzed by ClockLab software (Actimetrics).

### Adeno-associated virus production

AAV293 cells (Agilent Technologies) were cultured in 10 cm culture dish (1.25~1.5 × 10^6^ cells/dish) with 10 mL culture medium (DMEM (Sigma-Aldrich, D5796-500ML), 10% FBS (GE Life Sciences, HyClone SH30396.03), Penicillin Streptomycin (gibco, 15140-122), GlutaMax (gibco, 35050-061)). After 2~3 days, cells were passaged into T-150 flasks (2.5~3.0 × 10^6^ cells/flask) with 35 mL of culture medium. After 48~72 h, when cells reached 60~70% confluency, 25 mL of culture medium was removed, and 9 mL of fresh culture medium was added. Subsequently, plasmids were transfected via calcium phosphate co-precipitation. Briefly, three plasmids—pAAV-DJ, pHelper, plasmid including genes of interest—were added to 6 mL of 0.3 M CaCl_2_. Next, 6 mL of 2x HBS (280 mM NaCl, 1.5 mM Na_2_HPO_4_, 50 mM Hepes) was added with thorough mixing. 3 mL of the mixed compound was applied to each flask and further cultured for 6~12 h. Next, the culture medium was replaced with 35 mL of medium containing trichostatin A (DMEM, 2% FBS, Penicillin, Glutamax, 100 nM trichostatin A (Wako)), and further cultured for 48~96 h. Subsequently, the medium containing scraped cells was collected and underwent freeze-thaw three times. Finally, 1/10,000 volume of 250 U/µL TurboNuclease (Accelagen) was added and incubated for 30 min at 37 °C. The AAVs were purified with ViraPur Kit (Virapur).

### Adeno-associated virus injection

We performed AAVs injection as previously described^52,53^. In brief, mice 6–8 weeks of age were stereotaxically injected. Animals were anesthetized with isoflurane (1–2%) and placed in a stereotaxic frame. The adeno-associated virus AAVDJ-CaMKII-iCre-T2A-mCherry (3.55~7.87 ×10^11^ vg/mL) or AAVDJ-CAMKII-mCherry (7.83 × 10^12^ vg/mL) were injected to the olfactory bulb or the PC bilaterally. For AAV transduction to the granule cell layer of the olfactory bulb, we injected 300 nL of AAV to each olfactory bulb bilaterally. For AAV transduction to the PC, we injected AAV to three coordinates to either side of the brain, with a total of six injection coordinates bilaterally with 300 nL injection volume per injection coordinate. For injection, we used a pulled glass capillary (Drummond) and injected through nanoliter pressure injection (Nanoject3, Drummond). Stereotactic injection coordinates to target the olfactory bulb or PC were obtained from the Paxinos and Franklin atlas (for olfactory bulb AP:4.2 mm ML:1.0 mm DV:1.0 mm from brain surface; for PC AP:1.8, 0.5, −0.5 mm ML:2.7, 3.5, 3.9 mm, DV:3.8, 3.8, 4.2 mm from brain surface). Animals were allowed to recover for at least 4 weeks before being used in experiments.

### Luciferase assay

PER2::LUC mice 4 weeks of age were used for luciferase assay. Mice were sacrificed, and dissected brains were immediately cooled in ice-cold Hank’s Balanced Sodium Saline. The piriform cortex and the SCN were further coronally dissected in 150μm-thickness with vibratome. Sectioned brain slices were placed on Milli Cell immersed in culture medium (5% MEM, 18% HBSS, 15 mM NaHCO3, 2 mM HEPES, 10 mM D-glucose, 1 mM L-glutamine, 175 mg/L Ascorbic Acid, 1 mg/mL insulin in 0.01 M HCl, 25% Horse Serum; pH adjusted to 7.0~7.4) with Luciferin, and incubated at 37 °C, 5% CO2. Luminescence was detected by Kronos Dio (Atto) with 1-min exposure, 10-min intervals. For tetrodotoxin application, Tetrodotoxin (Fujifilm) was applied to the culture medium with a final concentration of 20 µM.

### In silico data analysis

From the list of genes reported to show circadian fluctuations of mRNA levels in the SCN^36^, we manually searched the function of each gene via UniProt (https://www.uniprot.org/) and identified genes that were documented to be involved in neuronal processes.

### Real-time quantitative PCR

For RNA extraction, 8–10 weeks-old mice underwent a constant dark condition for 24–48 h. Brains were dissected, washed with HBSS, and immediately frozen with liquid nitrogen. RNA was extracted with RNeasy kit (Promega), and the cDNA library was created by first-strand reverse transcription with ReverTraAce Kit (Toyobo). For real-time quantitative PCR, cDNA samples were mixed with THUNDERBIRD SYBR qPCR Mix (Toyobo) and primers and underwent two-step PCR (95 ˚C 15 s→ 60 ˚C 60 s, 40 cycles).

Primer sequences for real-time quantitative PCR are explained in Supplementary Table 1.

### Statistics and reproducibility

The statistical significance between values were determined by one-way ANOVA, one-way ANOVA with Bonferroni correction, Wilcoxon rank-sum test, Wilcoxon rank-sum test with Bonferroni correction. All experiments were independently repeated two or more times.

### Reporting summary

Further information on research design is available in the Nature Portfolio Reporting Summary linked to this article.

## Supplementary information

Supplementary Information

Description of Additional Supplementary Files

Supplementary Data 1

Reporting Summary

## Data Availability

Source data underlying Figs. 1b, 1d–e, 2a, 2c, 3a–c, 3e, 4c, 4f–h, 5b–c, 6b–c, Supplementary Figs. 1b–d, 3b, and 4 are provided as Supplementary Data 1. All other data are available from the corresponding author upon reasonable request.
