# Supplementary material for: The circadian clock in the piriform cortex intrinsically tunes daily changes of odor-evoked neural activity
Source: Commun Biol. 2023 Mar 27;6:332. doi: 10.1038/s42003-023-04691-8 (PMC10043281; doi:10.1038/s42003-023-04691-8)
Supplement: Supplementary file 1 — Supplementary Information [file 42003_2023_4691_MOESM1_ESM.pdf]

Supplementary Information

The circadian clock in the mouse piriform cortex plays an intrinsic role in daily changes in odor-evoked neural activity

Shunsuke Takeuchi[1], Kimiko Shimizu[2,3], Yoshitaka Fukada[1, 3], and Kazuo Emoto[1, 4]

[1]Department of Biological Sciences, Graduate School of Science, The University of Tokyo, 7-3-1 Hongo, Bunkyo-ku, Tokyo 113-0033 Japan
[2]Department of Pathological Cell Biology, Medical Research Institute, Tokyo Medical and Dental University, 1-5-45 Yushima, Bunkyo-ku, Tokyo 113-0033 Japan
[3]Laboratory of Animal Resources, Center for Disease Biology and Integrative Medicine, Graduate School of Medicine, The University of Tokyo, Hongo 7-3-1, Bunkyo-ku, Tokyo 113-0033, Japan.
[4]International Research Center for Neurointelligence (WPI-IRCN), The University of Tokyo, 7-3-1 Hongo, Bunkyo-ku, Tokyo 113-0033 Japan

[*]Corresponding: emoto@bs.s.u-tokyo.ac.jp

SUPPLEMENTARY FIGURES

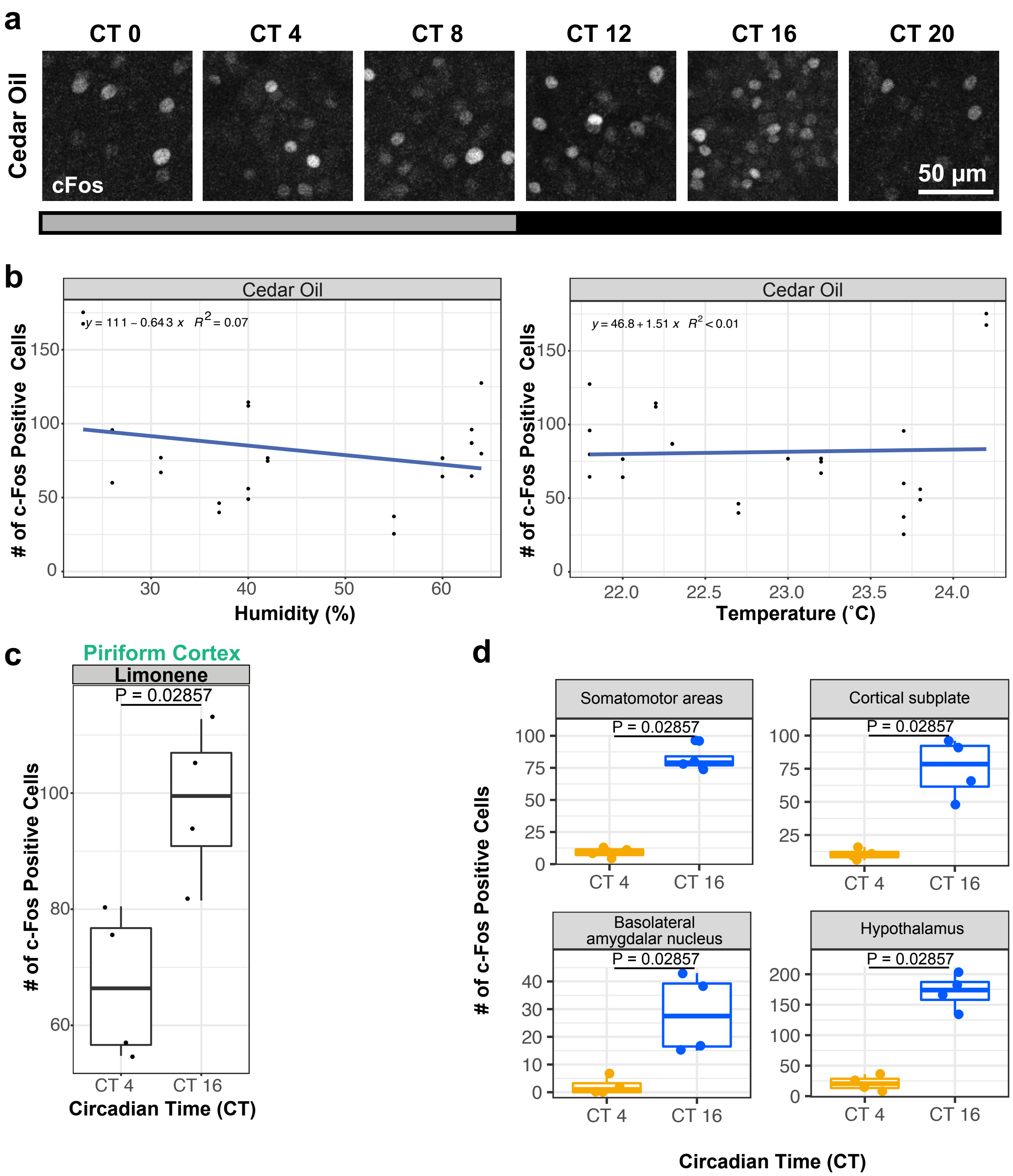

Supplementary Figure 1 Related to Figure1. Circadian Rhythm of Neural Activity in the PC

a Representative images of c-Fos expression in the PC of cedar oil presented group. scale bar = 50μm.

b Correlation coefficient of the number of c-Fos positive cells against humidity and temperature. R^2 = 0.07, $R^2 < 0.01$, respectively.

c Quantification of c-Fos positive cells in the PC of limonene-presented mice. 1,000-fold diluted limonene(-) in paraffin oil were presented for five minutes in this group. N = 4 for each timepoint, p = 0.02857, Wilcoxon rank-sum test. The line at the center of each boxplot depicts the median; the box depicts the third quartile and first quartile.

d Quantification of c-Fos positive cells in other brain areas: somatomotor areas, cortical subplate, basolateral amygdalar nucleus, hypothalamus. N = 4 for each timepoint, p = 0.02857, Wilcoxon rank-sum test. The line at the center of each boxplot depicts the median; the box depicts the third quartile and first quartile.

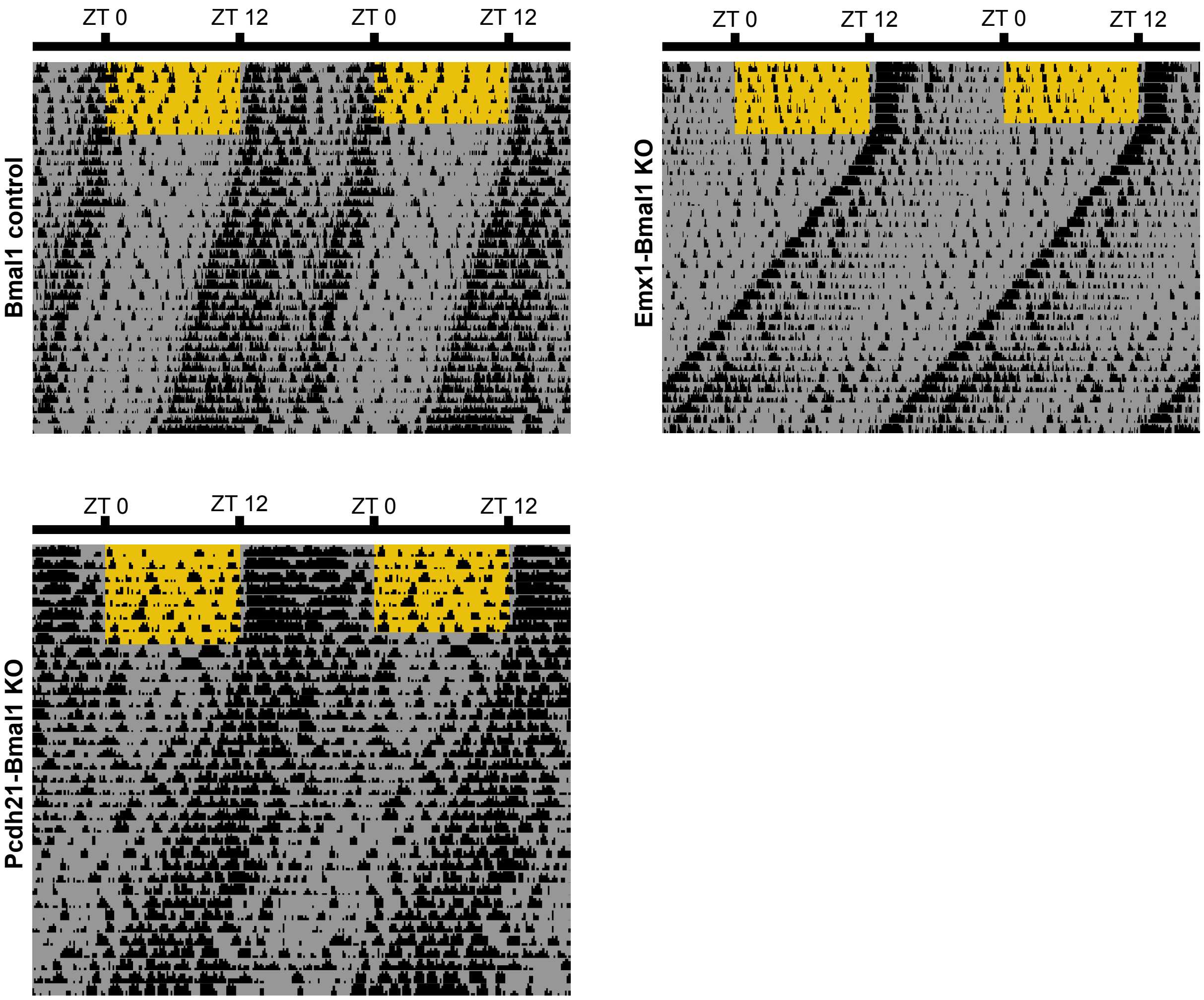

Supplementary Figure 2 Related to Figure 2. Locomotor Activity Patterns of *Bmal1* Conditional KO Mice

Locomotor activity of Bmal1-control mice, Pcdh21-Bmal1 KO mice, and Emx1-Bmal1 KO mice. Yellow shades and gray shades indicate light ON and OFF respectively.

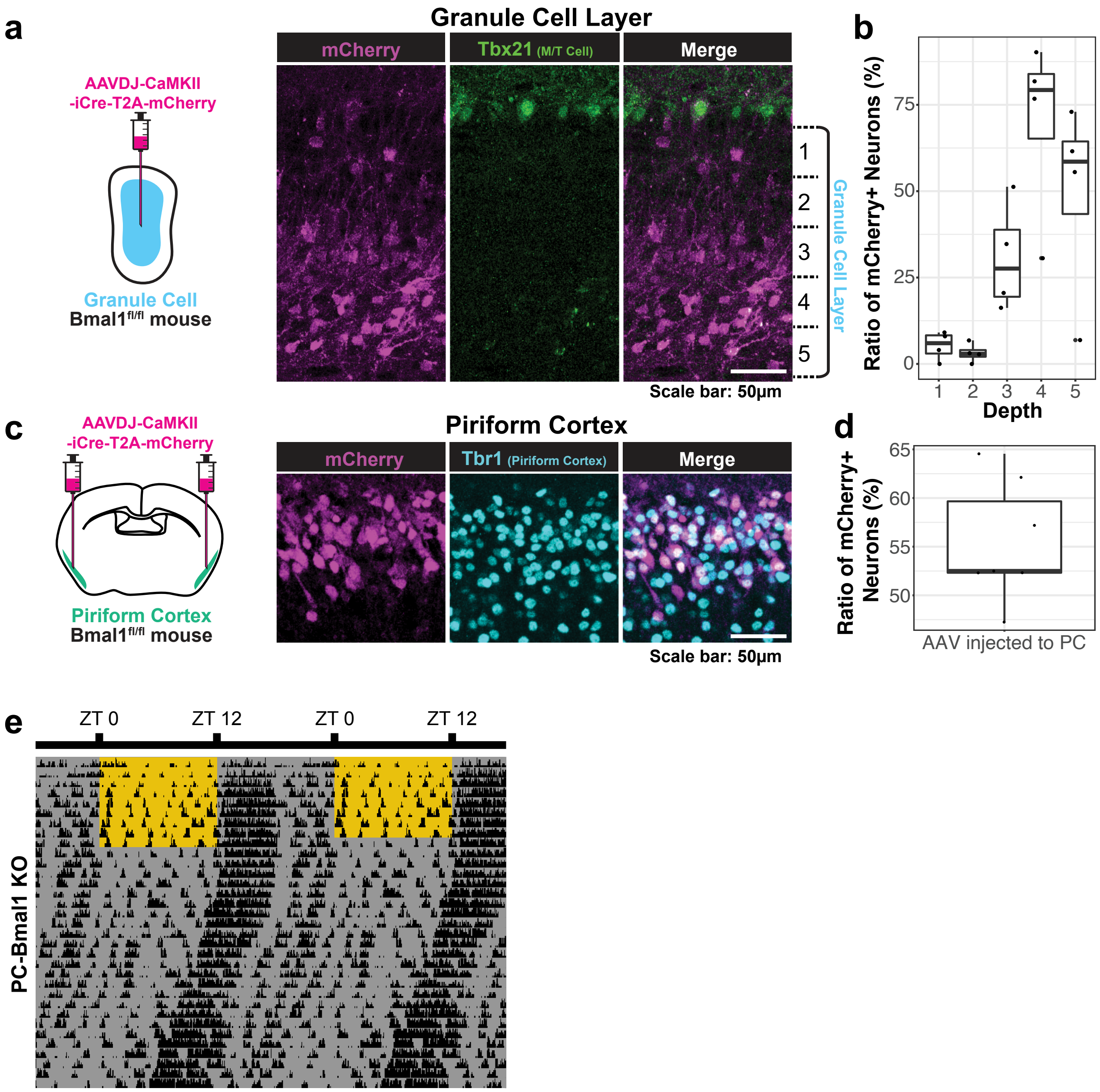
a
AAVDJ-CaMKII
-iCre-T2A-mCherry
Granule Cell
Bmal1fl/fl mouse
Granule Cell Layer
mCherry
Tbx21 (M/T Cell)
Merge
1
2
3
4
5
Granule Cell Layer
Scale bar: 50μm
b
Ratio of mCherry+ Neurons (%)
0
25
50
75
1 2 3 4 5
Depth
c
AAVDJ-CaMKII
-iCre-T2A-mCherry
Piriform Cortex
Bmal1fl/fl mouse
Piriform Cortex
mCherry
Tbr1 (Piriform Cortex)
Merge
Scale bar: 50μm
d
Ratio of mCherry+ Neurons (%)
50
55
60
65
AAV injected to PC
e
ZT 0
ZT 12
ZT 0
ZT 12
PC-Bmal1 KO

Supplementary Figure 3 Related to Figure3. Cre-Recombinase Expression patterns in Genetically Manipulated Mice

a Representative image of AAVDJ-CaMKII-iCre-T2A-mCherry injected to the olfactory bulb, stained with Tbx21 antibody. mCherry signals were observed in a subset of Tbx21- granule cells.

b The ratios of mCherry+ cells over Nissl+ cells were quantified in granule cell layers divided into five layers. 5.27 ± 4.14 %, 3.17 ± 2.80 %, 30.7 ± 15.8 %, 82.9 ± 6.82 %, 63.4 ± 8.85 % (mean ± standard deviation), in layers 1~5, respectively. The line at the center of each boxplot depicts the median; the box depicts the third quartile and first quartile.

c Representative image of AAVDJ-CaMKII-iCre-T2A-mCherry injected to the PC, stained with Tbr1 antibody. mCherry signals were observed in a subset of Tbr1+ PC neurons.

d The ratios of mCherry+ cells over Nissl+ cells were quantified in the PC. 55.6 ± 6.14 % (mean ± standard deviation). The line at the center of each boxplot depicts the median; the box depicts the third quartile and first quartile.

e Locomotor activity of PC-Bmal1 KO mice. Yellow shades and gray shades indicate light ON and OFF respectively.

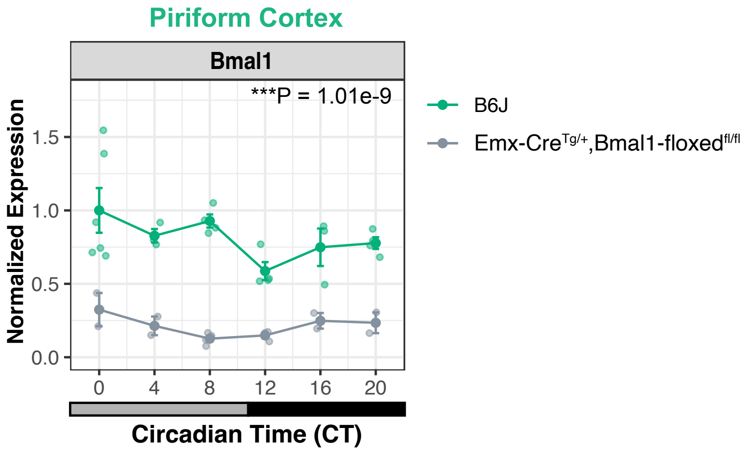

Supplementary Figure 4 Related to Figure6. Expression Patterns of *Bmal1* in Wild-Type and Emx1-Bmal1 KO Mice

Relative mRNA expression of *Bmal1* mRNA levels in the PC of wild-type mice and Emx1-Bmal1 KO mice. For wild-type mice, N = 6, 3, 4, 4, 3, 4 for CT0, CT4, CT8, CT12, CT16, CT20, respectively. For Emx1-Bmal1 KO mice, N = 2, 2, 4, 3, 2, 2 for CT0, CT4, CT8, CT12, CT16, CT20, respectively. p = 1.01e-9, two-way ANOVA followed by Tukey's post hoc test. Error bars indicate standard error.

Gene name	Forward primer (5’-3’)	Reverse primer (5’-3’)
Rps29	TGAAGGCAAGATGGGTCAC	GCACATGTTCAGCCCGTATT
Dbp	AATGACCTTTGAACCTGATCCCGCT	GCTCCAGTACTTCTCATCCTTCTGT
Bmal1	GCAGTGCCACTGACTACCAAGA	TCCTGGACATTGCATTGCAT
Rev-erba	CGTTCGCATCAATCGCAACC	GATGTGGAGTAGGTGAGGTC
Avpr1a	GCAGCGTGAAGAGCATTTCC	TCGGAATCGGTCCAAACGAAA
Clcn4	GCATTTAGAAGCACCACGCC	GGCAAGTGTTCAGCGTCATC
Chrnb2	TCCACTTGTGTTCCCTAGAAGA	GAGCCTCGCTGACACAAG
Snap25	TGGCTGATGAGTCCCTGGAA	CCATCCCTTCCTCAATGCGT
Gad1	AGGGATCGTGCAAGCAAGGAA	GTGGTCTTGGGGTCTCTACGG
Cacna2d3	CCATCCTGAGGAGAATGCAAGA	TCGCACCATAGTTGGGTTCA
Sst	CTGCGACTAGACTGACCCAC	CCAGTTCCTGTTTCCCGGTG
Syn1	ATCCCTGTCTCTGACCAATGC	TGTCAGTCGGAGAAGAGGCT
Calb2	TGATGCTGACGGAAATGGGT	TCCGCCATCTCAATTTTCCCA

Supplementary Table 1 Related to Figure6. Primers Used for RT-qPCR
