# Supplementary material for: The circadian clock in the piriform cortex intrinsically tunes daily changes of odor-evoked neural activity
Source: Commun Biol. 2023 Mar 27;6:332. doi: 10.1038/s42003-023-04691-8 (PMC10043281; doi:10.1038/s42003-023-04691-8)
Supplement: Supplementary file 2 — Description of Additional Supplementary Files [file 42003_2023_4691_MOESM2_ESM.pdf]

Description of Additional Supplementary Files

File name: Supplementary Data
Description: Source data underlying Figs. Fig 1b, 1d-e, 2a, 2c, 3a-c, 3e, 4c, 4f-h, 5b-c, 6b-c, Supplementary Figs. 1b-d, 3b, and 4.
