# Supplementary material for: The circadian clock in the piriform cortex intrinsically tunes daily changes of odor-evoked neural activity
Source: Commun Biol. 2023 Mar 27;6:332. doi: 10.1038/s42003-023-04691-8 (PMC10043281; doi:10.1038/s42003-023-04691-8)
Supplement: Supplementary file 4 — Reporting Summary [file 42003_2023_4691_MOESM4_ESM.pdf]

nature portfolio

Corresponding author(s): Kazuo Emoto

Last updated by author(s): Feb 12, 2023

Reporting Summary

Nature Portfolio wishes to improve the reproducibility of the work that we publish. This form provides structure for consistency and transparency in reporting. For further information on Nature Portfolio policies, see our Editorial Policies and the Editorial Policy Checklist.

Statistics

For all statistical analyses, confirm that the following items are present in the figure legend, table legend, main text, or Methods section.

n/a	Confirmed	
☐	☒	The exact sample size (*n*) for each experimental group/condition, given as a discrete number and unit of measurement
☐	☒	A statement on whether measurements were taken from distinct samples or whether the same sample was measured repeatedly
☐	☒	The statistical test(s) used AND whether they are one- or two-sided<br>*Only common tests should be described solely by name; describe more complex techniques in the Methods section.*
☐	☒	A description of all covariates tested
☐	☒	A description of any assumptions or corrections, such as tests of normality and adjustment for multiple comparisons
☐	☒	A full description of the statistical parameters including central tendency (e.g. means) or other basic estimates (e.g. regression coefficient) AND variation (e.g. standard deviation) or associated estimates of uncertainty (e.g. confidence intervals)
☐	☒	For null hypothesis testing, the test statistic (e.g. *F*, *t*, *r*) with confidence intervals, effect sizes, degrees of freedom and *P* value noted<br>*Give P values as exact values whenever suitable.*
☒	☐	For Bayesian analysis, information on the choice of priors and Markov chain Monte Carlo settings
☒	☐	For hierarchical and complex designs, identification of the appropriate level for tests and full reporting of outcomes
☒	☐	Estimates of effect sizes (e.g. Cohen's *d*, Pearson's *r*), indicating how they were calculated

Our web collection on statistics for biologists contains articles on many of the points above.

Software and code

Policy information about availability of computer code

Data collection	Fluorescent Images for immunohisotchemical analyses were obtained by KEYENCE or confocal microscopy Leica SP8.
Data analysis	R, ClockLab

For manuscripts utilizing custom algorithms or software that are central to the research but not yet described in published literature, software must be made available to editors and reviewers. We strongly encourage code deposition in a community repository (e.g. GitHub). See the Nature Portfolio guidelines for submitting code & software for further information.

Data

Policy information about availability of data

All manuscripts must include a data availability statement. This statement should provide the following information, where applicable:

- Accession codes, unique identifiers, or web links for publicly available datasets
- A description of any restrictions on data availability
- For clinical datasets or third party data, please ensure that the statement adheres to our policy

All data are available from the corresponding author upon request.

Field-specific reporting

Please select the one below that is the best fit for your research. If you are not sure, read the appropriate sections before making your selection.

☒ Life sciences ☐ Behavioural & social sciences ☐ Ecological, evolutionary & environmental sciences

For a reference copy of the document with all sections, see nature.com/documents/nr-reporting-summary-flat.pdf

Life sciences study design

All studies must disclose on these points even when the disclosure is negative.

Sample size	For immunohistochemical analyses, we chose the sample size n >2 according to the previous study using a similar experimental paradigm (Granados-Fuentes et al. 2006).<br>For luciferase assays, we chose the sample size n > 4 for each brain area according to previous studies (Yoo et al. 2004, Shimizu et al. 2016)<br>For RT-qPCR analyses, we chose the sample size n = 3-4 for each mouse genotype according to previous studies (Shimizu et al. 2016, Nakano et al. 2016).
Data exclusions	We excluded samples in which AAV transduction were unsuccessful. This was determined by the lack of fluorescent signal of mCherry.
Replication	All experiments were completed as multiple independent experimental runs, with a minimum of 3 replicate independent experiments.
Randomization	The experimetal approaches did not require samples to be randomized. Samples, and subsequent data collection and analyses were handled the same way in all experiments.
Blinding	For immnunohistochemical analyses, the genotype of the mice were blinded.

Reporting for specific materials, systems and methods

We require information from authors about some types of materials, experimental systems and methods used in many studies. Here, indicate whether each material, system or method listed is relevant to your study. If you are not sure if a list item applies to your research, read the appropriate section before selecting a response.

Materials & experimental systems

n/a	Involved in the study	
☐	☒	Antibodies
☐	☒	Eukaryotic cell lines
☒	☐	Palaeontology and archaeology
☐	☒	Animals and other organisms
☒	☐	Human research participants
☒	☐	Clinical data
☒	☐	Dual use research of concern

Methods

n/a	Involved in the study	
☒	☐	ChIP-seq
☒	☐	Flow cytometry
☒	☐	MRI-based neuroimaging

Antibodies

Antibodies used	anti-c-Fos (1:20000, Calbiochem, Cat#:PC38), anti-Tbr1(1:1000, Abcam, Cat#:ab31940), anti-BMAL1 (1:1000, Novus Biologicals, Cat#: NB100-2288), anti-Tbx21 (1:10000, gift from Y Yoshihara, (Mitsui et al. 2011)),
Validation	Validations of the primary antibodies are provided on the manufacturer's website

Eukaryotic cell lines

Policy information about cell lines

Cell line source(s)	AAV293 cells (Agilent Technologies
Authentication	*Describe the authentication procedures for each cell line used OR declare that none of the cell lines used were authenticated.*
Mycoplasma contamination	The cell line was not tested for mycoplasma contamination.
Commonly misidentified lines (See ICLAC register)	No commonly misidentified cell lines were used in this study

Animals and other organisms

Policy information about studies involving animals; ARRIVE guidelines recommended for reporting animal research

Laboratory animals	For immunohistochemical analyses and RT-qPCR analyses, Omp-Cre mice, Pcdh21-CreER mice, Emx1-Cre mice, and Vgat-Cre mice of C57BL/6J background were crossed with Bmal1-flox/xlox mice of C57BL/6J background.<br>For luciferase assays, PER2::LUC mice of C57BL/6J background were used.<br>Male mice were used in all experiments.<br>For immunohistohemical analyses and RT-qPCR analyses, mice were used at 8-12 weeks of age. For AAV injection experiment, we injected mice at 7-8 weeks of age. For luciferase assays, mice were used at 4 weeks of age.
Wild animals	N/A
Field-collected samples	N/A
Ethics oversight	Experiments were conducted in compliance with the rules and regulations established by the Animal Care and Use Committee of The University of Tokyo.

Note that full information on the approval of the study protocol must also be provided in the manuscript.
